# ‘We are All Interdependent’. A Study of Relationships Between Migrant Live-In Carers and Employers in Taiwan

**DOI:** 10.1177/23333936211043504

**Published:** 2021-10-31

**Authors:** Mai Camilla Munkejord, Tove Mentsen Ness, Wasiq Silan

**Affiliations:** 1 Department of Business Administration, Western Norway University of Applied Sciences, Bergen, Norway; 2 NORCE—Norwegian Research Centre, Bergen, Norway; 3 Faculty of Nursing and Health Sciences, Nord University, Namsos, Norway; 4 CEREN, Swedish School of Social Science, University of Helsinki, Helsinki, Finland

**Keywords:** Taiwan, elderly care, migrant care workers, live-in carers, quality of care, relations between care workers and employers

## Abstract

For the past three decades, to meet the increasing need for long-term care, the Taiwanese government’s primary approach has been to import migrant care workers. In this article, we analyse qualitative interview data produced in an Indigenous community. Drawing on Kittay’s feminist dependency theory, we explore the interrelationships and collaborative efforts between live-in carers and their employers. Three types of relationships were identified: ‘unsupportive relationships’, where the live-in carer was treated as a servant; ‘supportive relationships’, where the live-in carer was treated as a care worker; and ‘semi-supportive relationships’, where the live-in carer was treated as a carer-servant. In conclusion, the article sheds light on how the live-in carer arrangement could be practised in ways that allow live-in carers and thereby their care recipients to thrive.

## Introduction

Beliefs about how elderly care should be provided vary in different cultural settings. In Taiwan, as in most countries in Southeast Asia, providing long-term care for older family members has long been considered a private issue that should be handled within the household (Huang et al., 2012; Liang, 2014; Wang, 2010). The fact that Taiwan is a rapidly ageing society with a growing number of persons living with dementia coupled with the increasing number of women taking on paid work, however, has led to an explosion in the need for paid care services over recent decades (Chien, 2018; Liang, 2018). Nevertheless, partly due to the prevailing Confucian familistic ideology and reluctant politicians, professional home-based or community-based long-term care services have not yet been developed (Chien, 2018). Some nursing homes exist, but they are used to a very limited degree (Chou et al., 2015).

Since the early 1990s, as a result of national policies and bilateral migration agreements, recruitment agencies have imported migrant care workers to meet the increasing need for long-term care services in Taiwan. Although employing a migrant live-in carer is relatively expensive and inaccessible for poor and lower working-class families, the option is much used among families with sufficient means – even in Indigenous and rural parts of the country, as will be illustrated in this article. It should be noted that not only Taiwan but also most post-industrial countries actively recruit migrant care workers to address the issue of unmet long-term care needs (Atanackovic and Bourgeault, 2013; Cangiano and Shutes, 2010; Ho et al., 2019; Huang et al., 2012; Isaksen, 2010; Munkejord, 2016; Szeman, 2012; Tam et al., 2018). The cultural context and the working conditions that meet migrant care workers, however, vary considerably. Whereas migrant carers are primarily employed as professional care workers in the public (or private sector) in countries in Northern Europe and partly in Canada and the United States, in other parts of the world, such as southern Europe, the Middle East and Southeast Asia, including Singapore and Taiwan, migrant care workers are mainly employed as *live-in carers* in private households.

Thus, when a family in Taiwan is unable to cope with the care needs of older relatives, if they have the required resources, they would be expected to recruit a live-in carer. Indeed, the more intensive the care needs, the more likely it is for the family to employ a live-in carer (Chien, 2018, p. 1152). Thus, despite increasing regulations (Liang, 2015), the number of registered migrant care workers has reached more than 260 000 in 20201.

The live-in carers in Taiwan are all women and originate from a few selected countries, including Indonesia, the Philippines and Vietnam. Upon arrival, a number of restrictions are imposed on live-in carers, such as not having the right to stay for more than a predefined number of years and not having the right to change family unless the current employer agrees or the care recipient dies (Wang and Chan, 2017, p. 200). Moreover, as the Labour Standards Law does not apply to persons working in private households, live-in carers do not receive overtime payment, minimum wage, or paid days off (Liang, 2014, pp. 233, see also Chien, 2018). Additionally, while live-in carers according to the law are supposed only care for one person, in reality, they often must perform domestic chores for the wider household (Lan, 2003; Liang, 2011). This fact is underscored by recruitment agencies, which in their presentations of potential candidates provide very little information about workers’ qualifications in care work but rather emphasise candidates’ ‘willingness to do household chores and obey employers’ particular requests’ (Liang, 2011, p. 1824).

In the literature, live-in carers have often been portrayed as domestic workers subject to slave-like working conditions (Hoang, 2017; Lan, 2003; Parreñas, 2001). However, while the use of perspectives such as global care chains as theorised by Hochschild (2002), global servants as discussed by Parreñas (2001) or labour exploitation more generally (Kaur, 2010) may be crucial to shed light upon the precarious life conditions of millions of female migrant care workers worldwide, these perspectives fail to understand live-in carers as *care workers,* as well as the conditions under which the live-in carer arrangement may be practised in meaningful and supportive ways. In this article, we will address this knowledge gap by analysing qualitative interview data produced during the fall of 2019 in a mountainous Indigenous community in northern Taiwan. Drawing on feminist dependency theory (Kittay, 2011, 2020), we will explore the interrelationships and collaborative efforts between live-in carers and their employers. To conclude, this article aims to shed light on how the live-in carer arrangement could be practised in ways that allow live-in carers and thereby care recipients to thrive.

### Theoretical Perspectives on Caring as Relational (Inter) dependency

We all depend upon care at some points in our lives. Care receiving and caregiving, in other words, are central aspects of our being in the world (Fine and Glendinning, 2005; Kittay, 2011). According to Fine and Glendinning (2005), in sociology, care has mainly been studied in two ways: (a) by exploring informal caregiving as feminised, undervalued work and (b) by exploring the ethical and moral dimensions of care practices. The first body of literature understands care as a burden in terms of domestic duties and care tasks (Fine and Glendinning, 2005, p. 147). It typically addresses how gender and care are interrelated and the social implications of these interrelationships (Sigurdardottir and Kåreholt, 2014; Sihto, 2018). Additionally, this strand of research aims to reveal the hidden costs of informal care, as illustrated in an article by Munkejord et al. (2020). The second body of literature is more concerned with the moral and ethical dimensions of care. In this line of research, care as a practice is theorised through categories such as caring about, caring for, care giving and care receiving (Tronto, 2015). Additionally, although care in this research is also identified as a site of conflict, caring is primarily portrayed as a moral activity or as a moral concern for others (Fine and Glendinning 2005, p. 147–149)

Building on these bodies of research, the feminist philosopher Eva F. Kittay (2011, 2020) suggests a relational dependency approach to the study of caring. She terms care work as ‘dependency work’ and defines caregivers, whether they are paid or not, as ‘dependency workers’ (Kittay 2020, p. 3). The care recipient is termed the ‘charge’ (ibid). Care labour, Kittay notes, when it is well done, is characterised by ‘care, connection and concern’ (2020, p. 36). She clarifies that care labour as ‘care’ entails attending to others, for example, by giving medication, food and massage when the care recipient is in a state of vulnerability; care labour as ‘connection’ refers to the practices that sustain or create ties or intimacy between the dependency worker and the care recipient; and care labour as ‘concern’ refers to the affectional ties that sustain the connection. Care labour, Kittay (2011, p. 52) summarises, is thus about attending to the others’ needs in multiple ways and reflects a willingness to invest in the physical and emotional well-being of the other.

Importantly, however, if the dependency worker is to be capable of taking good care of the ‘charge’ (the care recipient), she also needs the required resources to be enabled to take care of her own well-being (2020, p. 46). These resources must be made available by ‘someone’ – whom Kittay terms the ‘provider’ (ibid). The relationship between the dependency worker and the provider is hierarchical (ibid). Whereas there may be economic, racial and/or gender dimensions, the main factors explaining this hierarchy are ‘the objective and subjective factors that make the exit option for the dependency worker less viable than those available to the provider’ (Kittay, 2020, p. 48). Moreover, the charge, the dependency worker and the provider all enter into dependency relations: the charge (the care recipient) and the dependency worker are in a relationship of ‘inevitable dependency’, whereas the dependency worker and the provider are in a relationship of secondary dependency. This means that as the charge depends on the dependency worker, the dependency worker herself depends on the provider (2020, p. 48). Importantly, and in line with Kittay (2020, p. 140), we argue that we should be aware of these double dependencies and seek to establish systems that take care of dependency workers in a way that allows them to ‘survive and thrive'.

## Methods

### Design and Study Setting

In this project, we wanted to better understand the phenomenon of live-in carers. Was it possible to identify live-in carers who experienced their life situation as meaningful, and if so what characterised the working conditions of these live-in carers? To explore these questions, a qualitative interpretative and constructivist research design was developed (Charmaz, 2006; Haavind, 1999). This approach assumes that our perceptions of reality are co-created. As this study is part of a project financed by the Research Council of Norway on ageing and care in Indigenous communities and since one of the members of the research team had grown up in a Tayal community in Taiwan, we decided to do this study among the Tayal, which is one of 16 Indigenous Peoples that has official recognition in Taiwan. The Tayal people count approximately 90,000.

### Sample and Recruitment of Participants

During our field trip in the fall of 2019, we were kindly invited to interview ten live-in carers and ten employers. All live-in carers and employers living in the Tayal community who volunteered to be interviewed were included in our study (convenience sampling). In nine of the ten families we visited, we also greeted the care recipient. In some cases, we also talked to other family members. The recruitment of participants went surprisingly smooth, primarily because the third author established cultural protocols and assisted in identifying gatekeepers who agreed to facilitate access to families with live-in carers in the community. This was done in three ways: First, via the third author, the research team was invited for coffee and inspiring and educational conversations with a Tayal family who had lived in the Tayal territory for many generations. Second, the research team was invited to visit three local Day Clubs for older people in the area. The Day Clubs were community-based care centres offering activities to promote healthy ageing in the area. Third, the third author and her parents introduced the research team to potential participants for the study. In these three ways, information about the project was quickly spread in the Tayal community, and within days, we were contacted by potential participants.

### Data Collection

The interviews were conducted as informal, semi-structured conversations. In five families, we did joint interviews in which the employer and the live-in carer were interviewed in the presence of each other, while in the other five families, we were allowed to interview the live-in carer (and thus the employer) separately. The live-in carers were asked about (a) their family and educational background, (b) their former work experience, (c) their everyday life as a live-in carer in the current family, (d) their relationships with their family in their country of origin and (e) their hopes and dreams for the future. The employers were asked about (a) when and how they decided to employ a live-in carer, (b) how it was to have a live-in carer in the family, (c) the tasks allocated to the live-in carer, and (d) how they would describe their relationship to their live-in carer (e.g. how did they communicate and how did they collaborate on care giving and household chores, if all all).

The data collection was performed by a team of two white, middle-class, middle-aged female established care researchers working in Northern Europe and a younger female Indigenous scholar of Tayal origin, who currently is living and working in Northern Europe. In the interview context, the first author introduced the study and led the interviews, while the second author listened and asked some questions at the end of each interview, and the third author translated the conversation (Mandarin Chinese – English). The recordings of each of the interviews lasted from 25 min to 2 h. The average was slightly more than 1 h. After each interview, the researchers wrote notes and reflections about the key themes emerging in the interview. All recordings were transcribed by the first author.

### Data Analysis

When analysing the data, we used a reflexive thematic approach (Braun and Clarke, 2006, 2019). In the following, we will explain how the analysis was performed to allow readers to assess the trustworthiness and relevance of the findings (Stige et al., 2009). First, the authors read the transcripts and the summaries of the interviews while noting themes that attracted our attention. Second, the research team met for a collaborative analysis workshop to discuss the data and potential analytical foci for our article (Braun and Clarke, 2006, 2019). We agreed that *the quality of the relationship* between the live-in carer and her employer seemed to be of particular importance to answer our research questions. All the cases, therefore, were thoroughly re-read examining how the live-in carer and the employer spoke about (a) their relationship with each other and related to that, (b) the tasks and responsibilities of the live-in carer and (c) the tasks and responsibilities of the employer or other family members with regard to household chores and care work. Eventually, three types of relationships were identified: unsupportive relationships, supportive relationships and semi-supportive relationships. Finally, we selected the cases that would be most suitable to illustrate the relationship types and, in addition, discussed which theoretical perspectives that would enable us to frame the findings in the best possible way. Kittay (2011, 2020) and her feminist dependency theory was chosen for several reasons. First, this perspective allowed us to view the work of live-in carers as *care labour* and not, for instance, ‘domestic work', as is often mistakenly the case in the literature. Second, Kittay’s perspective allowed us to illustrate that care work is not only a set of techniques or practices but also entails connection and concern for the care recipient. Third, and most importantly, Kittay and her feminist dependency theory helped us to illustrate how the quality of care provided to the care recipients (a) is inherently relational and (b) seems to depend on the quality of the relationship between the carer and her employer.

### Ethical Considerations

As mentioned above, the care recipients were present in all but one case. The researchers tried to be sensitive to the situation in each household and tried our best not to be disruptive. Therefore, one interview with one live-in carer was ended earlier, as we saw that the care recipient was becoming increasingly restless. We also tried to be as quiet as possible in cases where the care recipient slept during the interview. Moreover, in a couple of cases where the live-in carer was occupied and had limited time to speak with us, we asked only the most important questions.

Moreover, in five of the ten cases, the employer chose to be present during our interview with the live-in carer. In these cases, we tried to be sensitive to the ambiance in the home, and when we felt that the employer was somewhat defencive, we avoided asking questions that could possibly harm the relationship between the live-in carer and the employer. In a couple of families, however, we sensed, to our surprise, that the employers’ presence was not defencive, but rather supportive for the live-in carer, helping her to answer our questions. It should also be noted that we identified both supportive and unsupportive relationships both in joint interviews and in interviews where employers and carers were interviewed separately.

We would like to emphasise that ethical challenges are integral to all studies of vulnerable migrants (van Liempt and Bilge, 2012). Live-in carers are in an exposed position, whether they are interviewed alone or together with their employer. This is because as migrants working in people’s homes, they are tied not only to potentially exploitative employers but also to unfair legislations and potentially exploitative recruitment agencies. In this study, we acknowledge these ethical challenges and try to overcome them by making the research process as transparent as possible.

Formal ethical permission to undertake this study was obtained from the Norwegian Centre for Research Data. Before interviewing employers and live-in carers, oral consent was obtained. To protect the participants’ identities, no real names were used in the transcripts. Instead, we used general titles and identification numbers such as live-in carer 1, employer 1 and live-in carer 2. All transcripts were stored securely in password-protected files.

## Results

### Brief Presentation of Participants

The ten employers had between 1–14 years of experience with a live-in carer in their family. Eight of the employers were of Tayal origin, but Mandarin Chinese was the language most commonly spoken in all interviewed families. In terms of economic status, they varied from well-off middle-class families with good pensions to vulnerable working-class families. The live-in carers were from Indonesia (9) and the Philippines (1), which reflects the fact that practically all live-in carers in the region where this study was conducted originated from Indonesia. The live-in carers ranged from 25 to 45 years of age and had from three to more than 15 years of professional experience in Taiwan and/or other countries, such as Saudi Arabia, the United Arab Emirates and Hong Kong. All of them returned home to visit their family in their country of origin every three to 5 years, mostly only for a few weeks at the time. The majority of the live-in carers were the ‘first daughter’ of poor families, having grown up with the responsibility of providing for their parents and younger siblings.

In the following, we present the three types of live-in carer-employer relationships identified in our analysis: unsupportive relationships, supportive relationships and semi-supportive relationships. The person receiving care, which Kittay (2011, 2020) terms ‘the charge’, is referred to with the terms ‘care recipient’ or ‘Grandpa’/‘Grandma', the latter of which were terms used by the live-in carers as a sign of respect. It should be noted that in the first and second cases presented below (characterised by unsupportive and supportive relationships, respectively), the live-in carer and the employer were interviewed together, whereas in the third case (characterised by a semi-supportive relationship), the participants were interviewed separately.

### Unsupportive Relationships: The Live-in Carer as a ‘Servant’

In some of the families we visited, the live-in carer was considered a ‘servant’ by their employer. The live-in carer was expected to perform all the care tasks both day and night, as well as all household chores such as laundry, cooking and cleaning without assistance from family members. The employers in these families sometimes expressed that they had expected even more help from their live-in carer, despite a strict and packed timetable. The live-in carers employed in these families found it hard not being allowed to be in regular contact with their own family, not being able to sleep uninterruptedly through the night, and not receiving any help with either care work or house chores from family members.

A case that may illustrate the category of ‘unsupportive relationships’ is a household consisting of a male care recipient, his wife and a live-in carer who had worked for them for less than a year. When we arrived for the interviews on a brisk and sunny day in November, we could smell a hint of urine in the living room. The sofa was covered by towels. The care recipient, a Grandpa with dementia in his 90s, was seated in a chair, while the live-in carer, a young woman with a mild and docile appearance, gave him a banana. The employer, who was the care recipients’ wife, said that her relationship with the current live-in carer was ‘ok’, but that she sometimes shouted at her, admitting that she had ‘a hot temper'.

The employer told us that she met her husband, a war veteran who was more than twice her age at the time, *via* a friend of her father. She described her marriage as ‘without romance', adding: ‘That is how it was done at that time. I married him to help my family’. The couple had settled in the village because of his work. They had four children. Some of the children currently lived nearby, but they rarely came home to visit because they were ‘working a lot’.

The employer reported that before the live-in carer arrived, absolutely all the tasks in the household were on her shoulders, in addition to the increasing care tasks for her husband who suffered from dementia. After years like that, she could no longer cope. Her children had helped her with the application process, but the wife paid for the expenses herself with her husband’s pension. The first live-in carer had left for another family shortly after her arrival. The current live-in carer was their second one. She had several years of experience both with families in the Middle East and with another family in Taiwan. The live-in carer had to do all the house chores, in addition to assisting Grandpa both day and night. The employer explained:

Therefore, in the morning, when (the live-in carer) wakes up, she will mop the floor and do the laundry. And, then, at seven o’clock, when he (the husband) wakes up, she will make milk for him, and give him milk and bread.

In addition to having to do all the cleaning and cooking, and in addition to helping Grandpa with all the meals, the live-in carer had to give Grandpa massage and help him exercise twice a day, and, in addition, during the night, she had to help him to pee in a bottle every 3 h.

The live-in carer was married and had a small child in Indonesia. When we asked her if she kept in touch with her family by video calls, as was common among most of the live-in carers we interviewed, she answered hesitantly, looking shyly at her employer: ‘Not every day. Every 2 or 3 days. Or… (short silence) I don’t talk so much with them. I send text messages, and that is good for me because I am working here’. The employer, who listened attentively to our conversation with the live-in carer, confirmed that ‘the live-in carer is in this family to *work*', and she added that in her opinion, the live-in carers earned good money and had very good working conditions, at least if you compared her to Indonesians working in the local hotel business.

To summarise, in the households placed in the category ‘unsupportive relationships', the live-in carer was expected to do all the practical care work both day and night, in addition to the laundry, cooking and cleaning in the household. In these families, the live-in carer was talked about as a servant who received little, if any, assistance from the employer or other family members. Often, the live-in carer had to follow a strictly defined and packed timetable. In these households, we met exhausted live-in carers who felt overwhelmed by the tasks and duties she was expected to perform and who, due to sleep deprivation, were not necessarily able to attend to the care recipient in anything other than a mechanical, practical manner. This challenging work situation for live-in carers is in line with what has often been identified in research about live-in carers to date (Chien, 2018; Huang et al., 2012; Liang, 2014; Wang, 2010)

### Supportive Relationships: The Live-In Carer as a ‘Care Worker’

In other families, we identified a more supportive relationship between the employer and the live-in carer. These families were often middle-class with available economic and social capital and wanted to treat the live-in carer as a ‘care worker', Thus, although the work schedule was tight and although the live-in carers rarely had a day off, they told us that they had time to rest a little bit every day, and they had time to communicate with their own family via different digital platforms on a regular basis. Additionally, the live-in carers in these households felt that the employer was supportive and enabled them to do a good job. A case that may illustrate the category of supportive relationships is a large household consisting of a care recipient, several brothers and sisters and their (nearly) adult children, and the live-in carer. When arriving in this household, the research team was warmly welcomed by the employer, who accompanied us into the separate apartment where the care recipient, Grandma, lived together with the live-in carer. The live-in carer was married in Indonesia and had two children that she had not raised herself. At the time of the interview, her own children were already adults, and she was looking forward to becoming a grandmother. And now; a glimpse from our visit in this family:

Grandma, the care recipient, was sitting in her wheelchair in a nice dress with the live-in carer at her side, smiling. The tea was ready to be served. The employer told us that Grandma had suffered a serious cerebral stroke many years ago. While the first live-in carer had run away after only some months, the current care worker had stayed for more than ten years, only interrupted by two short visits to her family in Indonesia. The employer explained that Grandma had adult children who did not live in Taiwan and that while one of Grandma’s children covered the salary for the live-in carer, the employer and the other siblings covered the costs for accommodation, food, medication, clothes, and other necessities. The employer told us that a family member used to come to pray and have a cup of tea with Grandma and the live-in carer in the morning, and in the evenings, another family member often helped Grandma video call one of her children.

The live-in carer, who told her story with the employer still present in the apartment (the employer having an encouraging tone vis à vis the live-in carer), said that in the beginning, caring for Grandma had been quite challenging: ‘Grandma was truly violent, she would grab me and bite me’, she said. As the years passed, however, Grandma had calmed down, but it continued to be difficult to persuade Grandma to take her medication. The live-in carer explained: ‘If I speak in a normal tone she won’t listen. I have to raise my voice, and I have to repeat, and *then* she will follow’. When we asked the live-in carer about her daily routines, she said:

I wake up in the morning at half past six, and then organise everything, and at half past seven, I will wake up Grandma. Today, I helped her take a shower, and I washed her hair, and then I gave her breakfast... She sometimes eats a bun or toast, and she drinks milk.

Researcher: And then after breakfast, what do you normally do?

She will watch TV, and sometimes I wash clothes or the floor, or I will be in the back doing stuff – and then nothing special... (smiling).

The live-in carer added that she used to put Grandma to bed around six in the evening, and after that, the time was ‘her own’. The employer explained that the live-in carers’ schedule was flexible and that the most important thing was to care for Grandma. Unlike many other families, the employer added, she did not want the live-in carer to clean other places aside from where the care worker and Grandma lived. Additionally, the employer did not expect the live-in carer to take care of any other person in the family but Grandma. The employer was very happy with the live-in carer saying that ‘The live-in carer is a *great* person. She is *very patient*. Helping (Grandma) is not easy. Sometimes Grandma is angry, and it may be difficult…’ The employer added that she totally trusted the live-in carer to take good care of Grandma, explaining:

We Indigenous peoples, we treat live-in carers as our family. Because after all, they come all the way here to earn money, to provide for their children, so we need to treat them well, we cannot treat them as servants. So, we are different from other ethnic groups (in Taiwan), probably because of our religion, because the Christian religion has taught us that no matter where you are from, you should be treated as part of the family, that *we are all the same*. (…) We need to love.

In summary, in the households placed in the category ‘supportive relationships', the employer considered the live-in carer as a *care worker* who had come, first and foremost, to be a good and safe companion for a frail grandparent. Sometimes, the live-in carers in these families were asked to cook or to do the laundry, but the care tasks were to be prioritised. In addition, in the families characterised by a supportive employer–live-in carer relationship, the employer and other family members would assist the live-in carer in different ways, for instance, by allowing her to rest and allowing her to be flexible and to follow the care recipients’ rhythm throughout the day rather than having to follow a very strict schedule. Moreover, in these families, the live-in carer was talked about in a friendly and respectful manner as someone the employer trusted to make good decisions for the care recipient. This kind of relationship has rarely been identified in research so far.

### Semi-Supportive Relationships: The ‘Carer-Servant’

In some families, the situation was somehow in between the two cases presented above: the live-in carer was considered a *carer-servant* who was expected to do most of the care work and household chores, but she was supported by household members within their (limited) abilities. The households placed in this category in our study were working-class families with less available social and economic capital, and the care recipient in these families had intensive care needs.

A household that may illustrate this category consisted of the care recipient – a Grandma living with advanced dementia – and two adult children – a daughter and a son, both divorced with no children, and, for the past months, the live-in carer, a woman of approximately 40 with an adult child in Indonesia and many years of experience as a live-in carer. Grandma had no pension, so her adult children both needed and wanted to work to earn enough money for their daily living. In this case, we interviewed the live-in carer and the female employer separately: the live-in carer was interviewed at home over a cup of green tea. During the interview, Grandma was wandering restlessly around in the living room. The employer was interviewed a couple of days later at her business.

As in the supportive case referred to above, also this employer trusted and spoke well of her live-in carer, but the home situation was more complicated. First, the family lived in a small house located along a narrow and steep road where it was impossible for Grandma to walk. Therefore, Grandma and the live-in carer were always forced to stay inside the house or in the small backyard. These constraining living conditions sometimes triggered challenging behaviours in Grandma, such as frustration, aggression and restlessness, both night and day. Second, as both adult children in the household needed to work, the live-in carer was alone with her charge for 12 hours every day. Third, Grandma was not able to watch TV or do other activities on her own, and she needed to be attended to at all times. Working as a live-in carer in this household, therefore, was very demanding.

The female employer explained that her mother had been transformed from an active and independent older woman to a person in need of care 24/7 in only a couple of years. Finding a good live-in carer that her mother liked, turned out to be difficult. First, they received a temporary live-in carer:

Oh, that process was painful (…). My mother criticised the live-in carer, saying that she was ugly, that she had an ugly smile… So, it was difficult for her to take care of my mom, since my mom was criticising and insulting her all the time.

After the temporary live-in carer left, the daughter asked an aunt to help. That did not work well, either: the aunt did not understand Grandma’s condition and tried to reason with her, which led to them quarrelling and insulting each other all the time. Then, a new live-in carer arrived, but she did not want to stay long. According to the employer, she was ‘fearful’, and in addition, she hardly understood a single word of Mandarin Chinese. It was therefore difficult to communicate, and the employer simply had to accept that she wanted to leave after a short period of time. Finally, the current live-in carer arrived. The employer had been both nervous and excited at her arrival, hoping it would work this time. Luckily, it did:

So, for the current live-in carer (…), the first impression was good. (…) As she has a lot of experience already, in the beginning, I just observed a little bit on the side how she interacted with my mom, and then, after a couple of days, I just let her do her stuff. Also, I observed that the energy between the live-in carer and my mom was good.

Grandma even gave the live-in carer her own mother’s name, since they were both ‘very beautiful, very gentle and hardworking’. When we asked the employer about the live-in carers’ daily tasks, she explained that there were no specific routines. Everything was up to the live-in carer to decide depending on the mother’s condition and her wishes that day. The live-in carer confirmed this, explaining that whenever Grandma woke up in the morning, she would help her shower and give her breakfast, and other than that, the activities were up to them. The live-in carer explained that she used to prepare dinner for Grandma, the employers and herself every evening and that normally, she found some time to do the laundry or mop the floors. The one most important task, however, was to be a good and safe companion for Grandma:

Live-in carer: The most important is to ensure that Grandmother is taken well care of. Because my employer does not really intervene. (…) So, if Grandma wants to sing, we sing, and if she wants to dance, we will dance.

Researcher: Right. What kinds of songs does she remember? What language are they in?

Live-in carer: The only songs that we both know are in Japanese. Otherwise, we sing separately the same melodies each in our own language.

Although the live-in carer and Grandma often had a good time while singing and playing, caring for Grandma was challenging at times. The live-in carer explained, tears in her eyes:

Sometimes it is very difficult to care for Grandma. Grandma suddenly gets angry, she suddenly insults you, but when she does that, I hide away. I try to ignore her. And when she calms down, I will get closer to her again. Because of her disease, it is like that. I also need to prevent her from biting me. So, we should not get too close. Otherwise, it is fine.

Grandma used to wake up several times every night wanting to get up. However, in this household, the male employer, and not the live-in carer, was the one who slept next to Grandma and tried to calm her down during the night. The live-in carer, on the other hand, was invited to sleep in a different part of the house with the female employer. Allowing the live-in carer a good night’s sleep was probably required to enable her to thrive in an otherwise quite challenging care situation.

In households with semi-supportive relationships between live-in carers and employers, live-in carers had to do most of the care work and household tasks. The employers said that they would have wanted to assist the live-in carer more, but lacked the monetary or human resources to do so.

## Discussion

To date, there has not been much research on the relations between employers and live-in carers (Salami and Duggleby, 2017). Several studies, however, shed light on how employers perceive live-in carers. While some employers consider and treat them as a ‘care worker’, others consider and treat them as a ‘cook’, a ‘servant’ or a ‘domestic worker’ (Liang, 2017; Salami and Meherali, 2018; Wang, 2010; Wang and Lin, 2012). In this study, we identified families where live-in carers were perceived and treated as a ‘servant’, families where live-in carers were treated as a ‘care worker’, and families where live-in carers were treated as a ‘carer-servant’. We noted that in the households treating the live-in carer as a ‘servant’, she was not supported or assisted by the employer but rather strictly surveyed and controlled. In the households treating the live-in carer as a ‘care worker’, she was supported and assisted by a trusting, collaborating employer, whereas in the households treating the live-in carer as a ‘carer-servant’, she was partly supported, but within the often quite limited capacities of the employer.

This study indicates that whether the employer–live-in carer relationship was supportive, semi-supportive or unsupportive seemed to depend primarily on the *employers’ attitudes or human values*, for example, whether the live-in carer, who most often comes from a poor background, was perceived as ‘someone equal’ or as ‘someone inferior’. Likewise, the quality of the relationship seemed to depend on the *employers’ expectations*, for example, the tasks that the live-in carer was expected to do and, related to that, what tasks the employer deemed the most important (e.g. domestic tasks, practical care or social/emotional care). Moreover, the quality of the relationship seemed to depend on the *health situation of the care recipient*: some care recipients were more challenging than others, and to be able to provide good care for these seniors and to avoid burn-out, the live-in carers needed moral and practical help from the employer and other family members, and importantly: time to rest. In all three types of relationships, our findings indicate that care work (a) is inherently relational and (b) seems to depend not only on the relationship between the live-in carer and the care recipient but also on the relationship between the live-in carer and her employer.

Our findings is supported by some previous studies indicating that the live-in carer-employer relationship does influence the well-being of live-in carers and, consequently, the quality of care offered to seniors (Ho et al., 2018; Tam et al., 2018). Tam et al. (2018), for instance, argue that receiving little or no support from the employer causes challenging working conditions and harsh living conditions for live-in carers, but it also reduces the well-being of the seniors being cared for. A study from Hong Kong, moreover, emphasised the importance of meeting the live-in carer with trust and regard and in viewing the migrant worker as a companion or care worker rather than as a domestic helper (Ho et al., 2018, p. 7). Another study from Hong Kong revealed that live-in carers ideally should provide emotional care, which is very challenging to do if carers are embedded in an oppressive relationship with a challenging care recipient or an unsupportive employer (Ho et al., 2019). Our study supports and extends these findings by suggesting that flexibility, trust, love and respect are fundamental to creating a supportive and healthy employer–live-in carer relationship. Moreover, a supportive employer–live-in carer relationship illustrates Kittay’s point that good care is not only about providing care as a set of techniques but also about connection and concern (2001, p. 52). This entails that the care worker should be *enabled by a supportive employer* to contribute to the *well-being* of the care recipient.

This study highlights the urgent importance of fostering a system that can help employers develop a supportive and trusting relationship with their live-in carer. This argument is supported by Kittay (2020, p. 140), who, with reference to the Greek word for service, *doulia*, states that ‘just as we require care to survive and thrive, so we need to provide conditions that allow others – including those who do the work of caring – to receive the care they need to survive and thrive’.

### Limitations

The data from our in-depth interviews allowed for a nuanced exploration of the live-in carer phenomenon and of how the live-in carer system could be practised in a more meaningful and sustainable way. However, as noted in the methods section, the employers decided to be present in half of the interviews with live-in carers. Their presence was experienced in different ways and affected what the live-in carers were able to share with us in various ways. In the first case presented in this article (an unsupportive relationship), the employer’s presence was experienced as limiting control. However, in the second case (a supportive relationship), the employer’s presence was experienced as encouraging support that helped the live-in carer speak her mind about both her current and former experiences. Additionally, it should be noted that since the live-in carers did not have any privacy or time off, we could not interview them unless we first obtained approval from their employer. Finally, we could add that we probably obtained access to ‘the better families’ in terms of employer–live-in carer relationships. Many live-in carers are probably exposed to even more demanding, violent and abusive conditions than what was found in this study. This assumption is supported by previous research that sheds light on the importance of developing policies and laws to provide incentives for treating migrant workers, including live-in carers in Taiwan and beyond, in a more just and more human way (Chen, 2016; Kaur, 2010).

## Conclusion and Implications for Nursing

Our study indicates that despite overwhelmingly negative findings in previous research on live-in carers, promising and supportive employer–live-in carer relationships where live-in carers thrive do exist. This article finds that employers’ positive and respectful attitudes towards the live-in carer, moderation in what tasks the live-in carer could be expected to do, and support with the various tasks that need to be done are crucial if one would like the live-in carer, and hence the care recipient, to thrive. As the live-in carers oftentimes perform quite complicated care tasks such as taking care of dementia patients and giving medications, professional nurses employed in hospitals or local health stations should supervise and cooperate with the live-in carers. Cooperation with skilled nurses will make the live-in carers more confident in their work, and thus enhance the quality of the services provided.

The live-in carer system will remain for many years to come both in Taiwan and in other parts of the world. Thus, to improve the working and living conditions of live-in carers and, is pivotal. Learning from the promising *live-in carer-employer* relationships presented in this article will bring us some steps forward in this regard, particularly at the micro level by highlighting the significance of the employer’s attitudes, expectations and practices. Because, as one of the Indigenous employers in our study said, ‘We are all interdependent. We need to love’.
